# Karl Stern (1906–1975)

**DOI:** 10.1007/s00415-014-7407-7

**Published:** 2014-06-17

**Authors:** Frank W. Stahnisch, Stephen Pow

**Affiliations:** Departments of Community Health Sciences and History, Hotchkiss Brain Institute and Institute for Public Health, University of Calgary, 3280 Hospital Drive N.W., Calgary, AB T2N 4Z6 Canada

**Keywords:** Dynamic psychiatry, Frankfurt am Main, History of neurology, Karl Stern, Montreal, Neuropathology, Twentieth century

## Abstract

The forced migration process of German-speaking neurologists and psychiatrists under the Nazis during the 1930s and 40s is often preoccupied solely with “successful” concepts and therapeutic approaches. The case of German-Canadian neurologist Karl Stern (1906–1975) is very instructive, however, since the process of forced migration, for him, proved to be a transitionary process from his former cutting edge work in neuropathology and holist neurology in Germany to clinical psychiatry and the development of the new discipline of geriatric medicine in Canada.


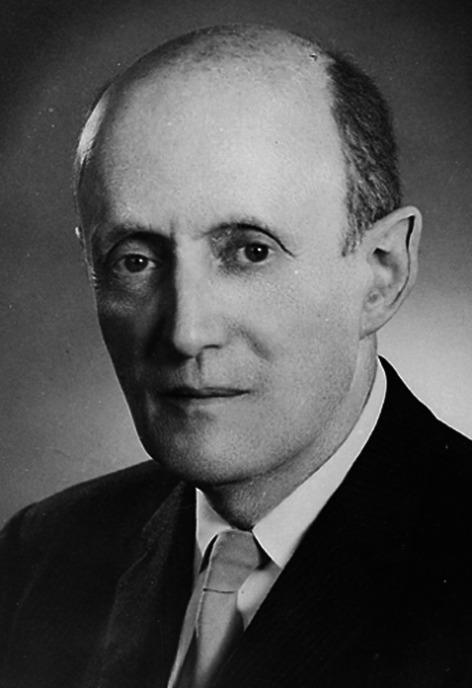
The historiography of the forced migration of European neurologists and psychiatrists under the Nazis, and other Fascist regimes of the 1930s and 40s, has often focused on the transfer of “successful” concepts and therapeutic approaches alone [1]. In the case of neurologist Karl Stern, it appears at first glance that the conditions for such a transfer of theories and methods were ideal [5]. Stern was born in the Bavarian town of Cham, near the Czech border. After completing his education at the Charité Medical School in Berlin, he graduated with a Dr. med. from the University of Frankfurt am Main in 1930. Between 1930 and 1931, he worked with holist neurologist Kurt Goldstein (1878–1965) at the Frankfurt *Institute for Research into the Effects of Brain Injuries* [7]. Then in 1932 he used a Fellowship from the American Rockefeller Foundation to conduct a neuropathological research project at the Munich-based German Research Institute for Psychiatry, where he collaborated with world-renowned tumor specialist Walther Spielmeyer (1879–1935). Stern obtained a position serving as Spielmeyer’s teaching assistant—a role that required enormous effort to live up to Spielmeyer’s high standard of expertise in neurodiagnostics. Graduate students and visiting fellows in Munich similarly came to expect the highest possible level of neurological education and training from Stern, whose clinical neuropathological work eventually would lead to an important publication appearing in the journal *Brain* in 1939: *Severe Dementia Associated with Bilateral Symmetrical Degeneration of the Thalamus*.

Stern’s collaboration with his other influential mentor, Goldstein, continued at the academic hospital of Berlin-Moabit, where he arrived in the latter half of 1932 to perform brain autopsies for Goldstein’s service [8]. The Moabiter Krankenhaus was then one of the few academic hospitals with services in neurology, psychiatry, and pathology that intricately related to each other along the same lines as formerly at the interdisciplinary Neurological Institute that Goldstein had directed in Frankfurt. However, the Nazi seizure of power drove both neurologists out of their academic positions, though Stern continued to work as a physician in private practice for another 2 years. In 1935, he decided to flee to London, where a tight network of contemporary scientists worked in his favor. The mentor from his Munich days, Walther Spielmeyer, had already familiarized Montreal-based neurosurgeon Wilder Penfield (1891–1976) with Stern’s work during a North American lecture tour in 1931 [10].

Following a period as a researcher in Penfield’s neuropathological service, Stern was recommended to psychiatrist D. Ewan Cameron (1901–1967). This recommendation became a decisive turning point in Stern’s career transition from strict neurologist to mental health expert with a public presence. Soon after the Allan Memorial Institute (AMI) opened in 1943, Stern began to work at the Geriatric Unit (the first in Canada) and taught courses as an assistant professor of psychiatry. However, Stern’s educational background had centered on a more encompassing approach to psychiatry and neurology, which proved quite different from Cameron’s biological orientation. Stern always referred back to his classical education in the German *Gymnasium* and the broad, humanistic learning of his medical studies, along with his immersion in Goldstein’s “holist neurology” [6]. Quite frequently the Sterns organized *soirées* at their home, inviting colleagues, academic friends, students, and interns to read psychoanalytic and philosophical works, or to listen to Karl’s recitals of Robert Schumann’s (1810–1856) piano pieces [2].

As the AMI developed into the leading Canadian center on biological psychiatry, Stern left Montreal in the 1950s to become a clinical professor of psychiatry at the new Ottawa Medical Faculty, where he took an overtly contrastive approach to psychiatry in his clinical and research program. While at McGill, he had already cultivated contacts in the francophone community of Québec from which a number of residents and interns joined his Geriatric Unit for individual study periods. Many of these psychiatrists also practiced psychotherapy or were trained as clinical psychoanalysts. When Stern assumed his professorship at the University of Ottawa, one of the French-trained psychiatrists, Victorain Voyer (1917–1975) followed him to his new department and helped to transform it into an important educational center for psychopathology and clinical psychiatry—one which would be closely associated with the Ottawa Mental Health Centre after 1961 [9]. In the same way that Stern had included basic psychoanalytic training into psychosocial research projects, he also introduced these modules into the psychoanalytic *Pavilion Albert Prévost* associated with the Université de Montréal, which he frequently visited over the years to deliver lectures and seminars [3].

Although the conditions for the transfer of ideas and methods had been ideal in Stern’s case, his biography cannot be regarded as a mere “success story” in terms of major theory-changes in the neurosciences: when Stern entered the neurosciences in Montreal, like other émigré physicians, he had to deal with the local research cultures; he did not, for the most part, manage to introduce his own holistic ideas of neurology. Achievements and institutional changes do not tell the full story however. There are numerous local accounts highlighting Stern’s ability as an influential academic teacher: he seemed to have interested a whole new generation of medical students in Montreal and Ottawa in a wide range of topics from the histological study of the brain, to psychopathology, to the anthropological perspective on psychiatry [4]. These accounts attest to the value of a broad training, which is often forgotten, owing to a sort of scientific tunnel vision that lauds scientific excellence in a specific discipline alone, while disregarding the broad education which history so often reveals as the potential source of future innovations.
